# Evaluation of Machine Learning Classifiers to Predict Compound Mechanism of Action When Transferred across Distinct Cell Lines

**DOI:** 10.1177/2472555218820805

**Published:** 2019-01-29

**Authors:** Scott J. Warchal, John C. Dawson, Neil O. Carragher

**Affiliations:** 1 Cancer Research UK Edinburgh Centre, MRC Institute of Genetics and Molecular Medicine, University of Edinburgh, Edinburgh, Scotland, UK

**Keywords:** high-content screening, cell-based assays, cancer and cancer drugs, machine learning

## Abstract

Multiparametric high-content imaging assays have become established to classify cell phenotypes from functional genomic and small-molecule library screening assays. Several groups have implemented machine learning classifiers to predict the mechanism of action of phenotypic hit compounds by comparing the similarity of their high-content phenotypic profiles with a reference library of well-annotated compounds. However, the majority of such examples are restricted to a single cell type often selected because of its suitability for simple image analysis and intuitive segmentation of morphological features. The aim of the current study was to evaluate and compare the performance of a classic ensemble-based tree classifier trained on extracted morphological features and a deep learning classifier using convolutional neural networks (CNNs) trained directly on images from the same dataset to predict compound mechanism of action across a morphologically and genetically distinct cell panel. Our results demonstrate that application of a CNN classifier delivers equivalent accuracy compared with an ensemble-based tree classifier at compound mechanism of action prediction within cell lines. However, our CNN analysis performs worse than an ensemble-based tree classifier when trained on multiple cell lines at predicting compound mechanism of action on an unseen cell line.

## Introduction

Cellular morphology is influenced by multiple intrinsic and extrinsic factors acting on cell physiology. Striking changes in morphology are observed when cells are exposed to biologically active small molecules. Compound-induced alteration in morphology is a manifestation of various perturbed cellular processes. We can hypothesize that compounds with a similar mechanism of action (MoA), which act upon the same signaling pathways, will produce comparable phenotypes, and that cell morphology can predict compound MoA. Multiparametric high-content imaging assays have become established across a number of screening groups to classify cell phenotypes from functional genomic and small-molecule library screening assays.^1^ The standard approach to extracting numerical features from cell morphologies is through the development and application of high-content image analysis algorithms, which segment cells and subcellular structures into “objects.” Then image-based measurements on those objects creates a multiparametric phenotypic fingerprint for each perturbation.^2–5^ Such methods are routinely applied to further evaluate the MoA of hit and lead compounds derived from conventional target-based drug discovery programs. This allows the use of more physiologically relevant cell-based assay conditions and also provides a phenotypic profile to help elucidate the MoA for hits discovered by target-agnostic phenotypic screening.^6^

A landmark paper in the field of high-content phenotypic profiling was published in 2004, when Perlman et al. first demonstrated that multiparametric phenotypic fingerprints could be clustered according to compound MoA using a custom similarity metric and hierarchical clustering.^2^ The majority of early high-content phenotypic profiling studies, utilizing morphological profiling, applied unsupervised hierarchical clustering in order to group treatments into bins that produce similar cellular phenotypes.^5,7^ More recently, several groups have evolved phenotypic profiling through the application of machine learning classifiers to predict the MoA of phenotypic hits, by comparing the similarity of the high-content phenotypic profiles with a reference library of well-annotated compounds.^4,8^ This can be performed by arranging unannotated compounds in feature space and using proximity to nearby labeled data to infer MoA.^4,9,10^ A slightly different approach is to train a classifier with labeled data and then attach labels to unknown compounds.^11,12^ However, the majority of such examples of compound MoA prediction are restricted to a single cell type, often selected because of its suitability for simple image analysis and intuitive segmentation of morphological features. The restriction of multiparametric high-content image analysis to single “easy-to-image” cell line models limits the application of phenotypic profiling and MoA classification studies across more morphologically complex and disease-relevant cell-based assay systems. Furthermore, the expansion of multiparametric high-content studies across broader panels of morphologically and genetically distinct cell lines, which more accurately represents the heterogeneity of human disease, has several benefits. This allows correlation of phenotypic response data with basal genomic, transcriptomic, or proteomic data to support further understanding of compound MoA at the molecular level and identification of biomarkers of phenotypic response. Such application of multiparametric high-content phenotypic screens across larger cell line panels, equivalent to the Cancer Cell Line Encyclopedia (CCLE) or Genomics of Drug Sensitivity in Cancer (GDSC) and new emerging induced pluripotent stem cell (iPSC)-derived model resources, can further support drug repurposing and pharmacogenomic studies across more complex cell-based phenotypes.

The aim of the current study was to evaluate the performance of a classic machine learning classifier applied to high-content morphological feature measurements and deep learning network classifiers applied directly to images. Our training and test datasets comprise an adaptation of a previously published cell painting assay^13,14^ (**Suppl. Table S1**) applied to eight genetically and morphologically distinct human breast cancer cell lines, representing four clinical subtypes (**Table 1**). Each cell line has been treated with 24 annotated small molecules representing eight therapeutic subclasses with the inclusion of two structurally distinct molecules for each subclass (**Table 2**). We present the results of compound MoA prediction across all eight breast cancer cell lines from our machine learning models using the following methods:

1. Ensemble-based tree classifier trained on extracted morphological features from five-channel images (CellProfiler)
2. Convolutional neural networks (CNNs) trained on five-channel images

**Table 1.** Panel of Breast Cancer Cell Lines Chosen for Study.

		Mutation Status	
Cell Line	Subclass	PTEN	PI3K
MCF7	ER	WT	E545K
T47D	ER	WT	H1047R
MDA-MB-231	TN	WT	WT
MDA-MB-157	TN	WT	WT
HCC1569	HER2	WT	WT
SKBR3	HER2	WT	WT
HCC1954	HER2	?	H1047R
KPL4	HER2	?	H1047R

PTEN = phosphatase and tensin homolog; PI3K = phosphoinsitide-3-kinase; ER = estrogen receptor; TN = triple negative; HER2 = human epidermal growth factor; WT = wild-type; ? = lack of consensus regarding the mutational status. The breast cancer cell line mutational status was taken from Dai et al.^19^

**Table 2.** Annotated Compounds and Their Associated MoA Label Used in the Classification Tasks.

Compound	Class	Subclass	Supplier	Cat. No.
Paclitaxel	Microtubule disrupting	Microtubule stabilizer	Sigma	T7402
Epothilone B	Microtubule disrupting	Microtubule stabilizer	Selleckchem	S1364
Colchicine	Microtubule disrupting	Microtubule destabilizer	Sigma	C9754
Nocodazole	Microtubule disrupting	Microtubule destabilizer	Sigma	M1404
Monastrol	Microtubule disrupting	Eg5 kinesin inhibitor	Sigma	M8515
ARQ621	Microtubule disrupting	Eg5 kinesin inhibitor	Selleckchem	S7355
Barasertib	Aurora B inhibitor	Aurora B inhibitor	Selleckchem	S1147
ZM447439	Aurora B inhibitor	Aurora B inhibitor	Selleckchem	S1103
Cytochalasin D	Actin disrupting	Actin disrupter	Sigma	C8273
Cytochalasin B	Actin disrupting	Actin disrupter	Sigma	C6762
Jasplakinolide	Actin disrupting	Actin stabilizer	Tocris	2792
Latrunculin B	Actin disrupting	Actin stabilizer	Sigma	L5288
MG132	Protein degradation	Proteasome	Selleckchem	S2619
Lacacystin	Protein degradation	Proteasome	Tocris	2267
ALLN	Protein degradation	Cysteine/calpain	Sigma	A6165
ALLM	Protein degradation	Cysteine/calpain	Sigma	A6060
Emetine	Protein synthesis	Protein synthesis	Sigma	E2375
Cycloheximide	Protein synthesis	Protein synthesis	Sigma	1810
Dasatinib	Kinase inhibitor	Src-EMT	Selleckchem	S1021
Saracatinib	Kinase inhibitor	Src-EMT	Selleckchem	S1006
Lovastatin	Statin	Statin	Sigma	PHR1285
Simvastatin	Statin	Statin	Sigma	PHR1438
Camptothecin	DNA damaging agent	Topoisomerase 1 inhibitor	Selleckchem	S1288
SN38	DNA damaging agent	Topoisomerase 1 inhibitor	Selleckchem	S4908

More specifically, we evaluate and quantify the performance of an ensemble-based tree classifier and a CNN classifier, with regards to predicting compound MOA, when trained and predicted on each individual cell line and on “previously unseen” additional cell types.

## Materials and Methods

### Cell Culture

The breast cancer cell line panel (**Table 1**) was grown in Dulbecco’s modified Eagle’s medium (DMEM; cat. 21969-035, Thermo Fisher Lifetech, Paisley, UK) and supplemented with 10% fetal bovine serum and 2 mM l-glutamine, incubated at 37 °C humidified and 5% CO_2_.

Cells were plated in 96-well optical bottom plates (cat. 165305, Thermo Fisher Lifetech, Paisley, UK) at a density of 2500 cells per well in 100 µL of media in the inner 60 wells and incubated for 24 h before compound treatment.

### Compound Treatment

Compounds (**Table 2**) were diluted in DMSO at a stock concentration of 10 mM. Compound plates were prepared in V-bottomed 96-well plates (cat. 3363, Costar, Bucks, UK) at 1000-fold concentration in 100% DMSO by serial dilutions ranging from 10 to 0.3 mM in semilog concentrations. We selected only three concentrations for this study as the most active concentrations (100, 300, and 1000 nM). Compounds were added to assay plates containing cells 24 h after initial cell plating and incubation by first making a 1:50 dilution in media to create an intermediate plate, followed by a 1:20 dilution from intermediate plate to the assay plate, with an overall dilution of 1:1000 from the stock compound plate to the assay plate.

### Modified Cell Painting Staining Protocol

To label cells in 96-well plates, the cells were fixed by adding an equal volume of 8% paraformaldehyde (cat. 28908, Thermo) to the existing media, resulting in a final paraformaldehyde concentration of 4%, which was left to incubate for 30 min at room temperature. The plates were then washed with 100 µL of phosphate-buffered saline (PBS) and permeabilized with 50 µL of 0.1% Triton X-100 solution for 20 min at room temperature. A solution of cell painting reagents was made up in 1% bovine serum albumin (BSA) solution (see **Suppl. Table S1**). Thirty microliters of cell painting solution was added to plates and left to incubate for 30 min at room temperature in the dark. Plates were then washed with 100 µL of PBS three times; after the final aspiration and addition of PBS, plates were sealed with a transparent plate seal (cat. PCR-SP, Corning, UK).

### ImageXpress Image Acquisition

Imaging was carried out on an ImageXpress micro XL (Molecular Devices, CA), a multiwavelength wide-field fluorescent microscope equipped with a robotic plate loader (Scara4, PAA, UK). In the cell painting assay used for this study, images were acquired in five fluorescent channels (as indicated in **Suppl. Table S1**) at 20× magnification; exposure times were kept constant between plates and batches as to not influence intensity values. Images were captured across four different sites per well, with each site containing approximately 50–200 cells in negative control wells depending on the cell line, with the MDA-MB-157 cell line containing fewer cells per image compared with the other tested cell lines due to the large cell size characteristics of that particular cell line.

### CellProfiler Image Analysis and Morphometric Feature Data Analysis

Images were analyzed using CellProfiler v2.1.1^15^ to extract morphological features. Briefly, cell nuclei were segmented in the Hoechst-stained image based on intensity, and clumped nuclei were separated based on shape. Nuclei objects were used as seeds to detect and segment cell bodies in the cytoplasmic stains of the additional channels. Subcellular structures such as nucleoli and Golgi apparatus were segmented and assigned to parent objects (cells). Using these masks marking the boundary of cellular objects, we measured morphological features for multiple image channels returning per object measurements. Out-of-focus and low-quality images were detected through saturation and focus measurements and removed from the dataset. Image averages of single-object (cell) measurements were aggregated by taking the median of each measured feature per image. Features were normalized on a plate-by-plate basis by dividing each feature by the median DMSO response for that feature; a z score was then calculated for each feature over the entire pooled dataset to standardize each feature to a mean of zero and unit variance. Feature selection was performed by calculating pairwise correlations of features and removing one of a pair of features that have a correlation greater than 0.9, and removing features with very low or zero variance of cellular objects.

### Ensemble Tree-Based and CNN Classifiers

The ensemble tree-based classifier was implemented using scikit-learn’s (version 0.19) “GradientBoostingClassifier” with default parameters except for the number of estimators, which was increased from 100 to 600. Data used for the tree-based classifier were median profiles representing an image average of the morphological features, and test accuracy was measured using the Jaccard similarity score.

The CNN classifier was implemented in PyTorch (version 0.3.0) by modifying the ResNet18 architecture^16^ to accept input in the form of five-channel arrays rather than the typical RGB three-channel images. The models were trained with batches of 32 images per graphics processing unit (GPU) with random 90° rotations for 20 epochs with an initial learning rate of 0.01 with an ADAM optimizer^17^ using categorical cross-entropy as the loss function; test accuracy was measured with the Jaccard similarity score.

The number of epochs was chosen based as the point at which training and validation accuracies plateaued, as well as losses stopped decreasing, when training and predicting MoAs on a single cell line (**Suppl. Fig. S1**).

Transfer learning was performed by training a CNN model on a dataset of seven cell lines, and then freezing the weights of the first six layers of ResNet18 to leave only the last convolutional block and the fully connected layer available for training, and then training on a small dataset of the unseen cell line with a reducing learning rate of 0.0001 for 30 epochs.

Images for the CNN model were created from five-channel fluorescent images by detecting nuclei locations in the Hoechst-stained image based on intensity and cropping the image in all five channels to a 300 × 300 pixel bounding box centered on the nuclei for each cell in the image. For CNN prediction, individual cells within each image were classified, and the most common classification for all the cells contained within an image was taken as the overall image classification.

When training and predicting on a single cell line, the single-cell image data were grouped by original image labels and randomly shuffled before splitting into 70% training and 30% test sets. Grouping by parent image was performed to avoid splitting cell images from the same parent image across training and test sets, which would lead to overfitting.

Class imbalance due to more training examples in certain MoAs, such as microtubule disrupters, was addressed by randomly undersampling overrepresented training set classes so that each training class contained a number of examples equal to the smallest class for that cell line.

Three different concentrations were used for each compound, and data relating to different concentrations were pooled and treated as a single class.

Transfer learning on a small subset of the data used a random 10% subset of the class-balanced dataset for each cell line, and testing on the original 30% withheld test set.

### Code and Data Availability

Python code and links to datasets are available at www.github.com/CarragherLab/2018-08_transfer_ML_between_cell_lines.

## Results

**Figure 1.**
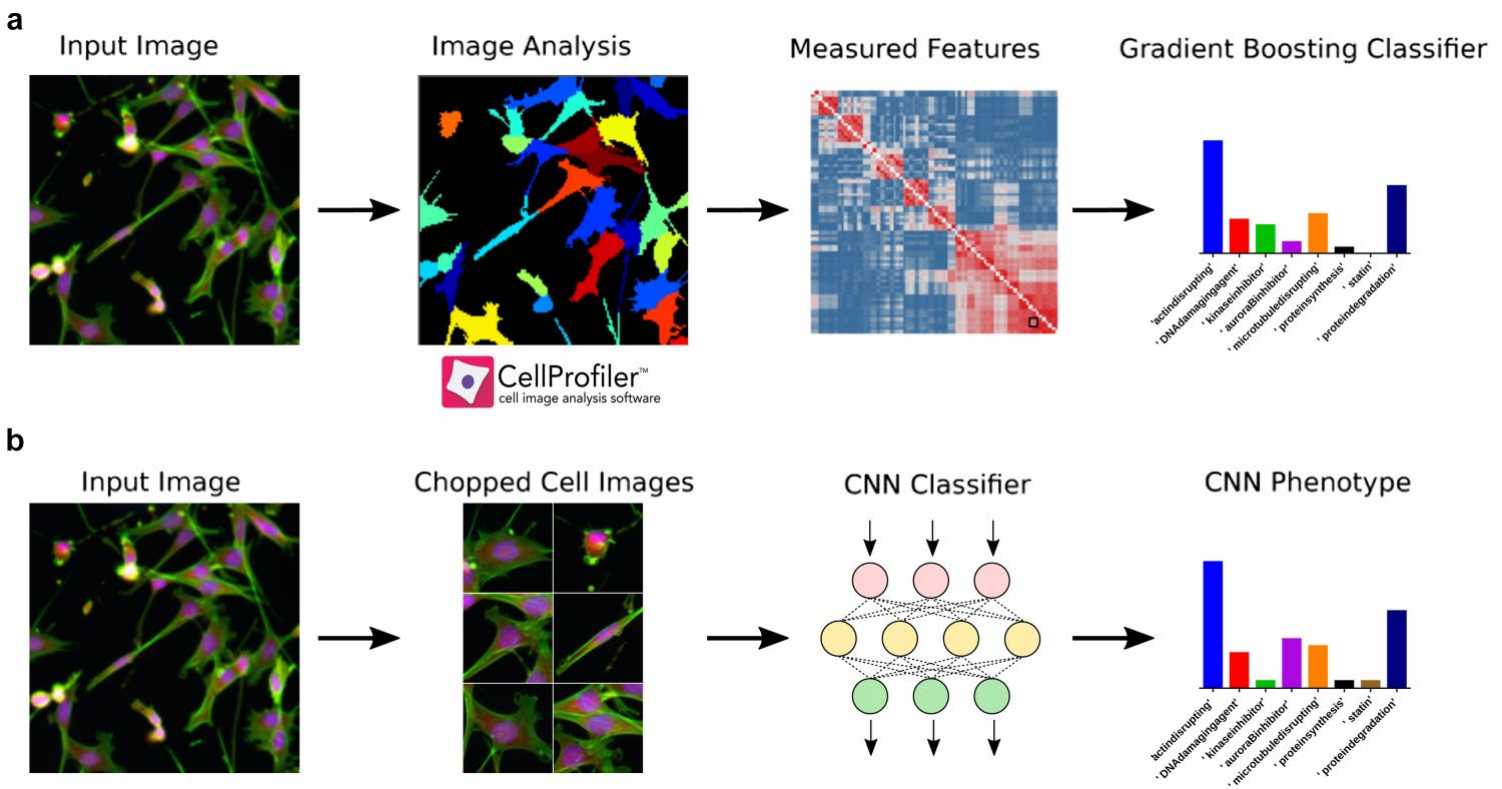
Workflow diagram. (**a**) Fluorescent cell images are segmented and morphological features are measured in CellProfiler, which are used in an ensemble-based tree classifier to train and predict compound MoAs. (**b**) Fluorescent cell images are chopped into 300 × 300 pixel regions around each cell and used as labeled input for a CNN classifier to predict compound MoA.

We present the results of comparing two methods for compound MoA prediction across eight breast cancer cell lines, treated with our 24-compound test set. We have used an ensemble-based tree classifier applied to image analysis-based measurements of morphological cell features and a CNN classifier applied directly to images. A schematic pipeline describing our profiling and classification strategy, using ensemble-based tree and CNN classifiers, to predict MoA across a panel of human breast cancer cells is presented in **Figure 1**.

**Figure 2.**
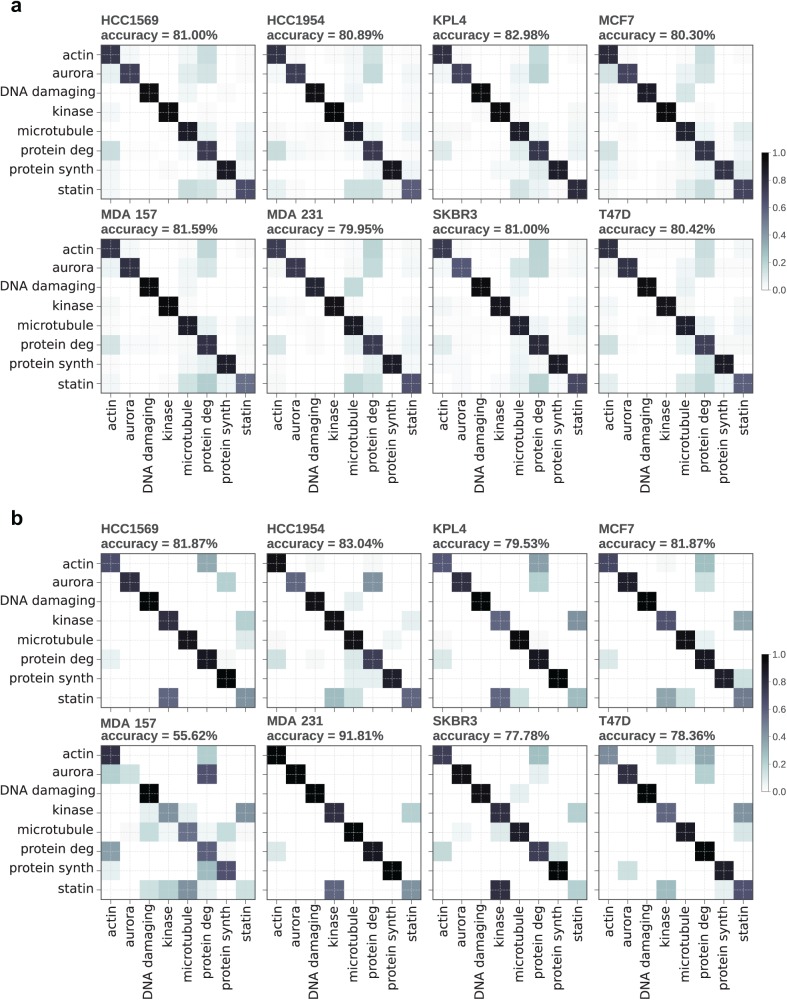
Confusion matrices for MoA prediction when trained and predicted on the same cell line. Data were split into 70%/30% training/test sets. (**a**) Ensemble-based tree classifier. (**b**) ResNet18 CNN classifier.

The results of the application of an ensemble-based tree classifier, trained on extracted morphological features from five-channel images (CellProfiler), for each individual breast cancer cell type, are presented in **Figure 2a**. The results of the application of a deep learning network (CNN), trained on five-channel images, for each individual breast cancer cell type, are presented in **Figure 2b**. These data demonstrate that when trained and predicted on the same cell line, an ensemble-based tree classifier and ResNet18 CNN classifier show generally equivalent performance in compound MoA prediction for the majority (seven out of eight) of cells. For one out of the eight cells (MDA-MB-157), the ensemble-based tree classifier outperformed the ResNet18 CNN classifier.

**Figure 3.**
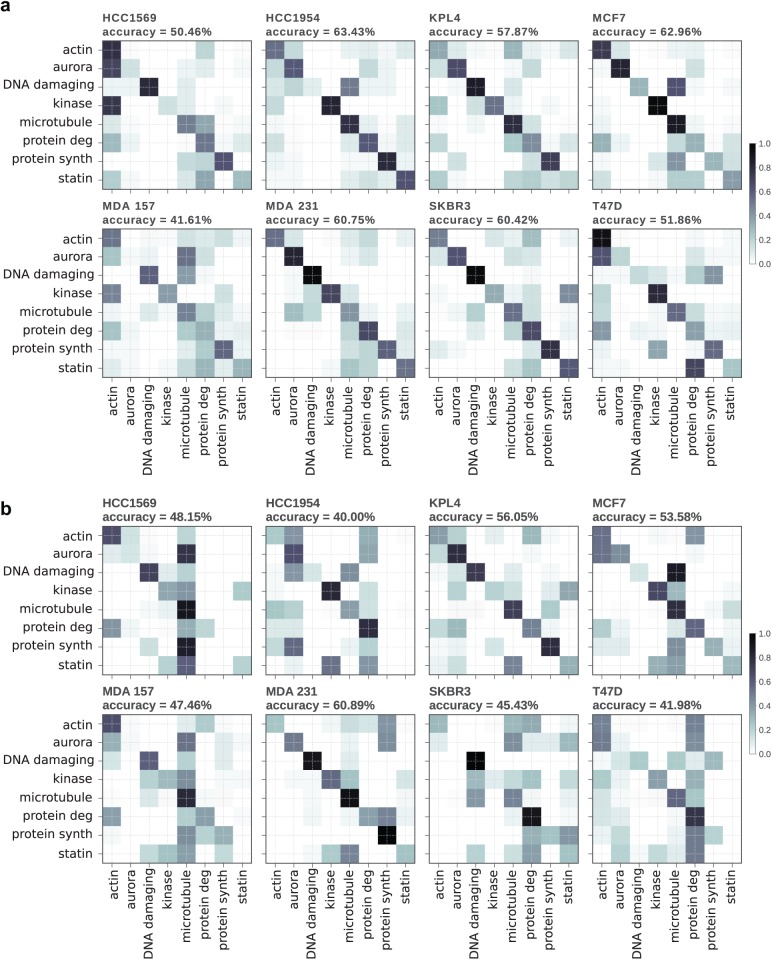
Confusion matrices for MoA prediction when trained on seven cell lines and tested on an unseen cell line. Titles indicate the unseen cell line. **(a**) Ensemble-based tree classifier. (**b**) ResNet18 CNN classifier trained on balanced class sizes by undersampling overrepresented MoA classes.

The predictive performance of the classifiers applied to unseen cell lines was compared by training on seven out of the eight cell lines and testing on the withheld eighth cell line. The ensemble-based tree classifier saw a reasonable reduction in classification accuracy when tested on unseen cell lines (**Fig. 3a**). The CNN classifier saw a dramatic decrease in prediction performance when trained on the original unbalanced dataset, caused by a strong bias toward MoA classes overrepresented in the training set, such as microtubule disrupters (**Suppl. Fig. S2**). Training the CNN classifier on a dataset in which the training examples contained equal numbers of each class greatly improved accuracy when predicting an unseen cell line. Accuracies were slightly below those of the tree-based classifier (**Fig. 3b**), although predictions still seem to be biased toward microtubule disrupters and prediction performance was decreased compared with training and predicting on the same cell line. It was found that applying the same undersampling balancing to the dataset used with the tree-based classifier greatly reduced prediction accuracies (**Suppl. Fig. S3**), likely due to the reduced number of training examples available.

Transfer learning was applied to the CNN models whereby models trained on seven cell lines were then trained on a small subset of the unseen cell line, with a reduced learning rate and frozen weights to stop the first six convolutional blocks from updating during training. This produced a large increase in predictive performance on the remaining test set of the unseen cell line (**Suppl. Fig. S4**), with accuracies similar to those of ResNet18 models trained and tested on the same cell line (**Fig. 2b**).

**Figure 4.**
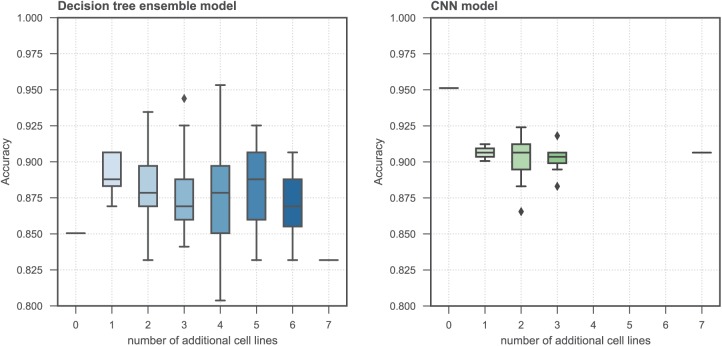
The effect of training with additional cell lines when predicting MoAs on a withheld 30% dataset of the MDA-MB-231 cell line. Box plots show testing accuracy when trained with different combinations of the additional cell lines.

In order to assess if training with additional data from morphologically distinct cell lines impacts model performance when predicting with one particular cell line, we trained both tree-based and CNN classifiers with 70% of the MDA-MB-231 dataset and combinations of additional cell line data and determined the prediction accuracy on the remaining MDA-MB-231 test data. It was found that with the tree-based classifier the incorporation of additional cell lines generally aids classification accuracy, although certain combinations decrease model performance below that of the baseline of just training and testing with the MDA-MB-231 cell line. On the contrary, CNN model performance decreased with the addition of further cell line data (**Fig. 4**). Due to the considerable compute time required to train CNN models, not all combinations of additional cell lines were assessed.

## Discussion

Predicting compound MoA from high-content image-based screening is a classification task, which can be approached in two ways. The first method is to develop an image analysis algorithm to extract morphological information from the images of cells and generate a multivariate dataset describing cellular phenotypes, which are subsequently used to train a classifier.^4^ The second approach is to use the image data as input in an appropriate classifier. Image classification has received lots of attention recently due to theoretical and technological breakthroughs. Such developments include the application of deep learning approaches incorporating neural network classification on raw image datasets. Artificial neural networks (ANNs) are becoming increasingly common in a wide range of machine learning classification tasks, and the convolution aspect of CNNs plays an important role when working with image data. CNNs have recently been applied to the classification of cell phenotypes from high-content imaging data, where they have been shown to perform well when using highly optimized publicly available high-content benchmarking datasets.^11^ Many groups have reported increased accuracy of CNN classifiers on high-content imaging data over approaches using extracted morphological features, which is in contrast to our own findings (**Fig. 2**).^9,10,12^ As this study was concerned with the generalizability of the classifiers across cell lines, we did not focus our efforts on absolute predictive performance, and we expect that further optimizations like those implemented by Ando et al.^9^ would increase prediction accuracy. It should be noted that when training and predicting on the same cell line (as in **Fig. 2** and **Suppl. Fig. S4**), a single dataset is split into training and test partitions. The method used in this study randomly sampled proportions, which may lead to overfitting and overoptimistic prediction accuracies due to the test data containing replicates from the same wells as found in the training set, as well as the same compound at different concentrations. Alternative approaches, which can be used in such instances, include a “hold one compound out” cross-validation strategy^4,9^ (see supplemental material describing the application of “leave one compound out” [LOCO] and “leave one compound and cell line out” [LOCACLO] out cross-validation methods applied to a subset of our data). As would be expected, using the LOCO and LOCACLO cross-validation strategies decreases prediction accuracies when compared with training and testing on random partitions of image data for both CNN and gradient-boosted tree classifiers. However, the cross-validation strategies mirror the results of the random partition of training and test method in that there was a significant loss of prediction accuracy when the CNN model was transferred to an unseen cell line (**Suppl. Figs. S5 and S6**).

Specific experimental questions addressed by this current study include direct comparison of the predictive performance between classical machine learning (ensemble-based tree classifier) and deep learning (CNN) models, when applied to predicting compound MoA across a disease-relevant panel of morphologically distinct human breast cancer cell lines. We further address how well the performance of each machine learning model can generalize to new cell line data. We trained our machine learning models on seven breast cancer cell lines and tested prediction accuracy on a withheld eighth cell line. Both types of classifier suffered a reduction in classification accuracy when applied to unseen cell lines, although the CNN performed noticeably worse on certain cell lines. This difference between classifier performance is likely explained by the data preprocessing steps in which the data for the ensemble tree-based classifier are subjected to plate-by-plate normalization to the negative control values, which will remove many of the cell line-specific morphologies, while this normalization step is not replicated in the CNN data preprocessing. As this is essentially an overfitting problem, there may be additional measures, such as further image augmentation, that may improve the generalizability of CNN models to distinct cell lines.^18^ The ability to predict compound MoA with a high degree of accuracy, despite the machine learning model never having seen this cell line before, will be very useful, for example, in cases where an investigator has trained a model on a large annotated compound set and would like to predict MoAs from further compound screens in new cell lines, without having to rescreen and retrain.

We also assessed if the addition of more data from morphologically distinct cell lines during model training increases prediction accuracy when applied to the classification of a single cell line. It was found that the CNN classifier did not benefit from the additional data, and that the possible benefit of more training examples did not overcome the increased heterogeneity and morphological differences between the cell lines. The ensemble-based tree classifier did generally benefit from additional training examples, although model performance was highly variable and sensitive to the combination of additional cell lines used during training. This leads us to the conclusion that more data are not necessarily better when training classifiers for MoA prediction, and those cell line-specific morphologies can dramatically impact model performance.

There are a number of reasons why transferring an MoA classifier from one cell line to another may fail. One possible explanation is that the intrinsic morphological differences between different cell lines are greater than the morphological changes induced by a compound, and that classifiers with no knowledge of cell line labels may confuse compound-induced morphologies with cell line morphologies. For example, if a cell line inherently has a large cell area morphology, it may often cause classification errors for compounds, which cause more spindle-like cell lines to spread. Alternatively, there may be a biological explanation, such as differential expression, mutation, or alternative splicing of pharmacological targets between the cell lines or altered downstream pathways, which may alter the phenotypic response of a cell line to a particular small molecule.

Our results indicate that while a CNN classifier may represent an efficient approach to study compound MoAs across larger cell line panels, the trained models are difficult to generalize between morphologically distinct cell lines. The more classical machine learning models trained on extracted morphological features offer opportunities to normalize the data in order to remove cell line-specific effects and improve model generalizability and transferability between cell lines. Broad cell panel screening studies that include cell line models presenting with more complex heterogeneous cell morphologies, including “clumpy” cell cultures, present significant challenges to efficient cell segmentation. The use of CNNs and the omission of custom image analysis algorithms for each cell line remove considerable resource burden and experimental or human bias when performing high-content imaging studies across morphologically distinct cell panels. Therefore, investigators need to weigh the pros and cons of each approach and its suitability to the dataset under investigation. Furthermore, the implementation of CNNs is likely to support the application of high-content phenotypic profiling and MoA classification across a broader variety of mechanistic classes and more complex assays, including iPSC differentiation, co-culture, and 3D models, which are often unsuitable for image-based segmentation. Alternatively, classical machine learning techniques, like ensemble-based tree classifiers, are much easier to interpret than CNNs, and inform which morphological features are important for distinguishing between mechanistic compound classes.

We anticipate that the methods and results presented in this article will support an increased prediction accuracy of compound MoAs across a broader variety of cell-based assay systems, including genetically distinct cell panels. Such approaches are well placed to advance in vitro pharmacogenomic studies beyond simple univariate assay endpoints toward more complex assays and phenotypes. Finally, we provide all source code and MoA datasets used in this study through a dedicated open-access link (www.github.com/CarragherLab/2018-08_transfer_ML_between_cell_lines). We anticipate that these data will support the high-content image analysis and machine learning communities, to further evolve new approaches to high-content phenotypic profiling and MoA prediction across morphologically distinct cell panels and more complex cell-based assay formats.

## Supplemental Material

Supplemental_Material_for_Evaluation_of_machine_learning_classifiers_by_Warchal_et_al-final-version3 – Supplemental material for Evaluation of Machine Learning Classifiers to Predict Compound Mechanism of Action When Transferred across Distinct Cell Lines Click here for additional data file. Supplemental material, Supplemental_Material_for_Evaluation_of_machine_learning_classifiers_by_Warchal_et_al-final-version3 for Evaluation of Machine Learning Classifiers to Predict Compound Mechanism of Action When Transferred across Distinct Cell Lines by Scott J. Warchal, John C. Dawson and Neil O. Carragher in SLAS Discovery
