# Supplementary material for: Evaluation of Machine Learning Classifiers to Predict Compound Mechanism of Action When Transferred across Distinct Cell Lines
Source: SLAS Discov. 2019 Jan 29;24(3):224–33. doi: 10.1177/2472555218820805 (PMC6484528; doi:10.1177/2472555218820805)
Supplement: Supplemental_Material_for_Evaluation_of_machine_learning_classifiers_by_Warchal_et_al-final-version3 – Supplemental material for Evaluation of Machine Learning Classifiers to Predict Compound Mechanism of Action When Transferred across Distinct Cell Lines [file Supplemental_Material_for_Evaluation_of_machine_learning_classifiers_by_Warchal_et_al-final-version3.pdf]

Supplemental Material

Evaluation of machine learning classifiers to predict compound mechanism of action when transferred across distinct cell-lines.

By Scott J Warchal, John C Dawson, Neil O Carragher

Cancer Research UK Edinburgh Centre, MRC Institute of Genetics and Molecular Medicine, University of Edinburgh, Edinburgh, Scotland, United Kingdom

Corresspondance:

Professor Neil Carragher, Cancer Research UK Edinburgh Centre, MRC Institute of Genetics and Molecular Medicine, University of Edinburgh, Edinburgh EH4 2XR, United Kingdom.

Email: n.carragher@ed.ac.uk

Keywords:

High content screening, cell-based assays, cancer and cancer drugs, machine learning,

Stain	Structure labelled	Wavelength (ex/em [nm])	Concentration	Cat. No., Supplier
Hoechst 33342	Nuclei	387/447	2 µg/mL	#H1399 Mol. Probes
SYTO14	Nucleoli	531/593	3 µM	#S7576 Invitrogen
Phalloidin-594	F-actin	562/624	0.85 U/mL	#A12381 Invitrogen
Wheat germ agglutinin-594	Golgi & plasma membrane	562/624	8 µg/mL	#W11262 Invitrogen
Concanvalin A-488	Endoplasmic reticulum	462/520	11 µg/mL	#C11252 Invitrogen
MitoTracker DeepRed	Mitochondria	628/692	600 nM	#M22426 Invitrogen

Table S1. Reagents used in the modified cell painting protocol and the excitation (ex)/emission (em) wavelengths of the filters used in imaging.

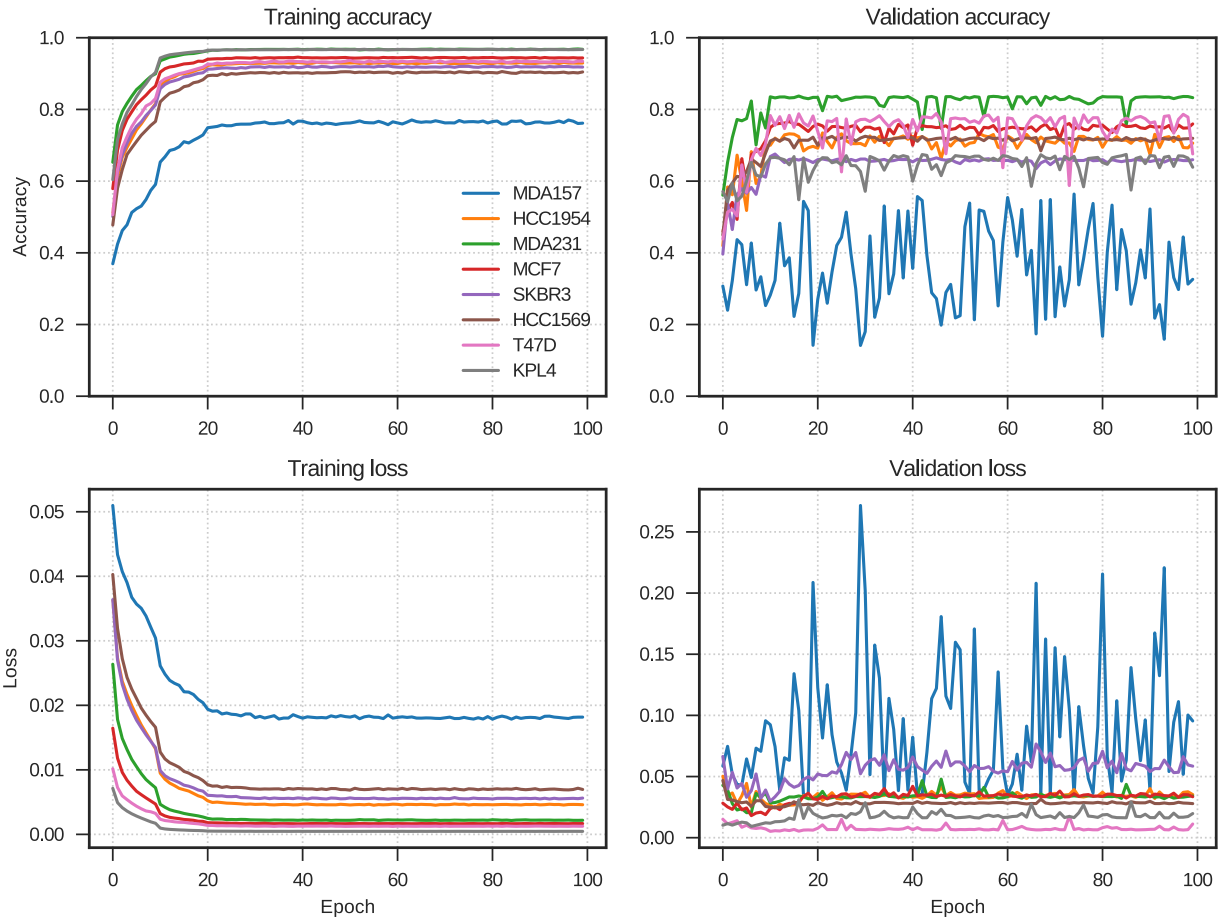

Figure S1: Accuracy and loss curves for ResNet18 training and predicting eight MoA classes on a single cell-line trained over 100 epochs. MDA 231: MDA-MB-231, MDA 157: MDA-MB-157

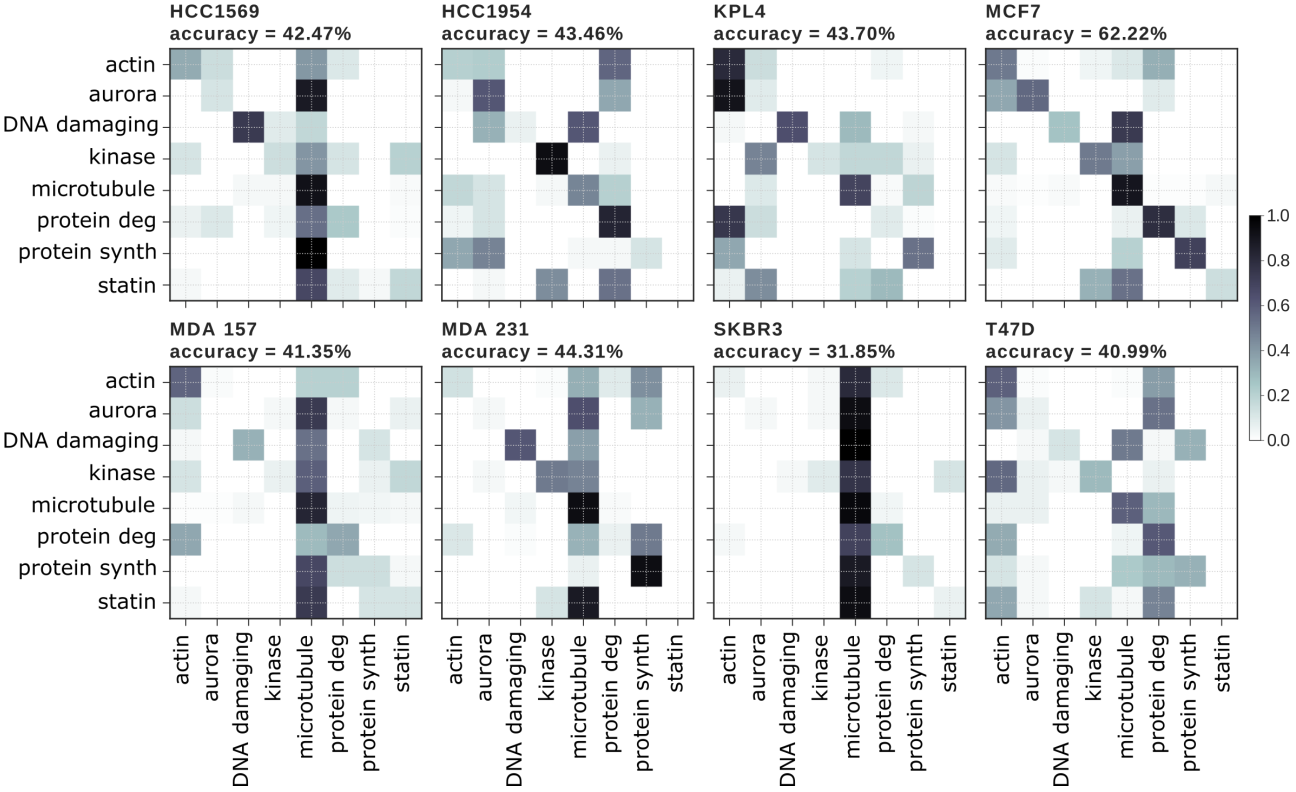

Figure S2: Confusion matrices for mechanism-of-action prediction using ResNet18 CNN trained on seven cell-lines and tested on an unseen cell-line. Training datasets were unbalanced containing differing number of training examples in each mechanistic class. Titles indicate the unseen cell-line.

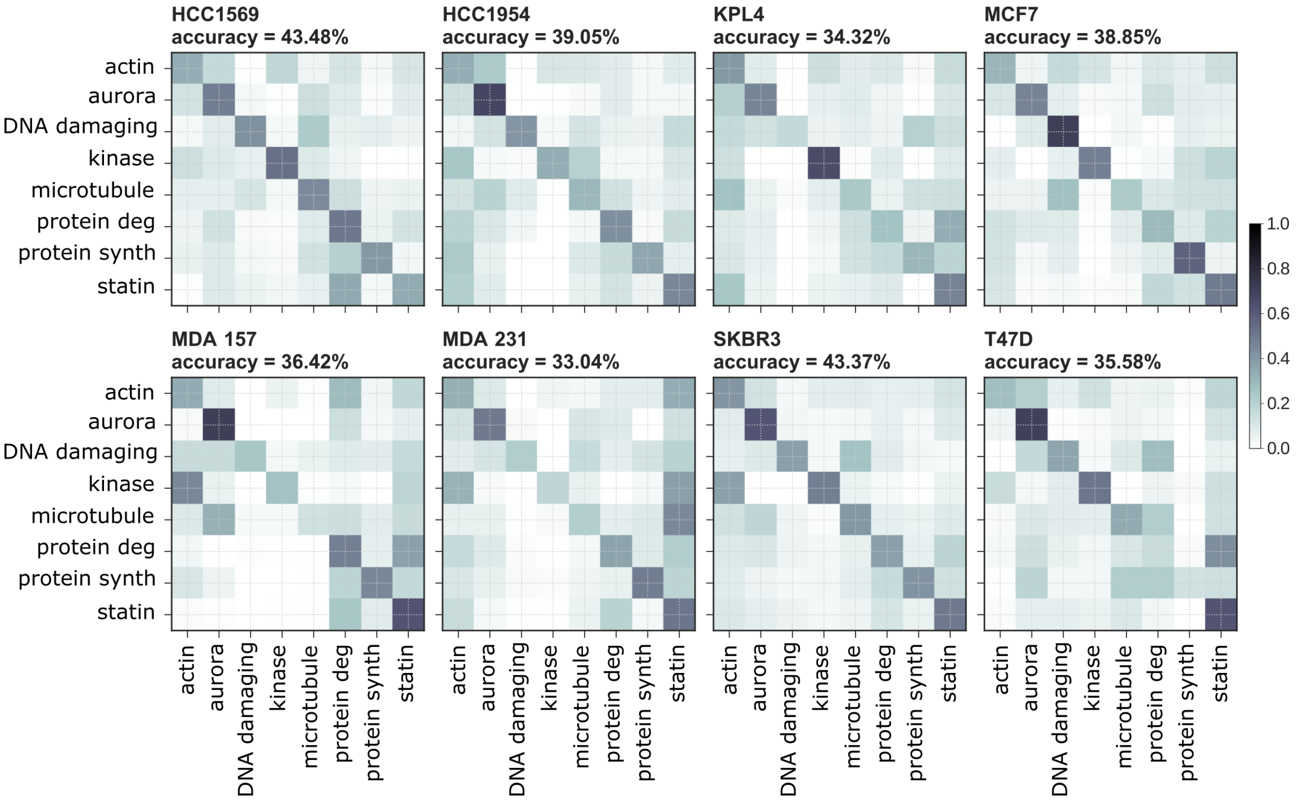

Figure S3: Confusion matrices for mechanism-of-action prediction using a gradient boosting tree classifier trained on seven cell-lines and tested on an unseen cell-line. Training datasets were balanced by undersampling over-represented classes to contain equal number of training examples in each mechanistic class. Titles indicate the unseen cell-line.

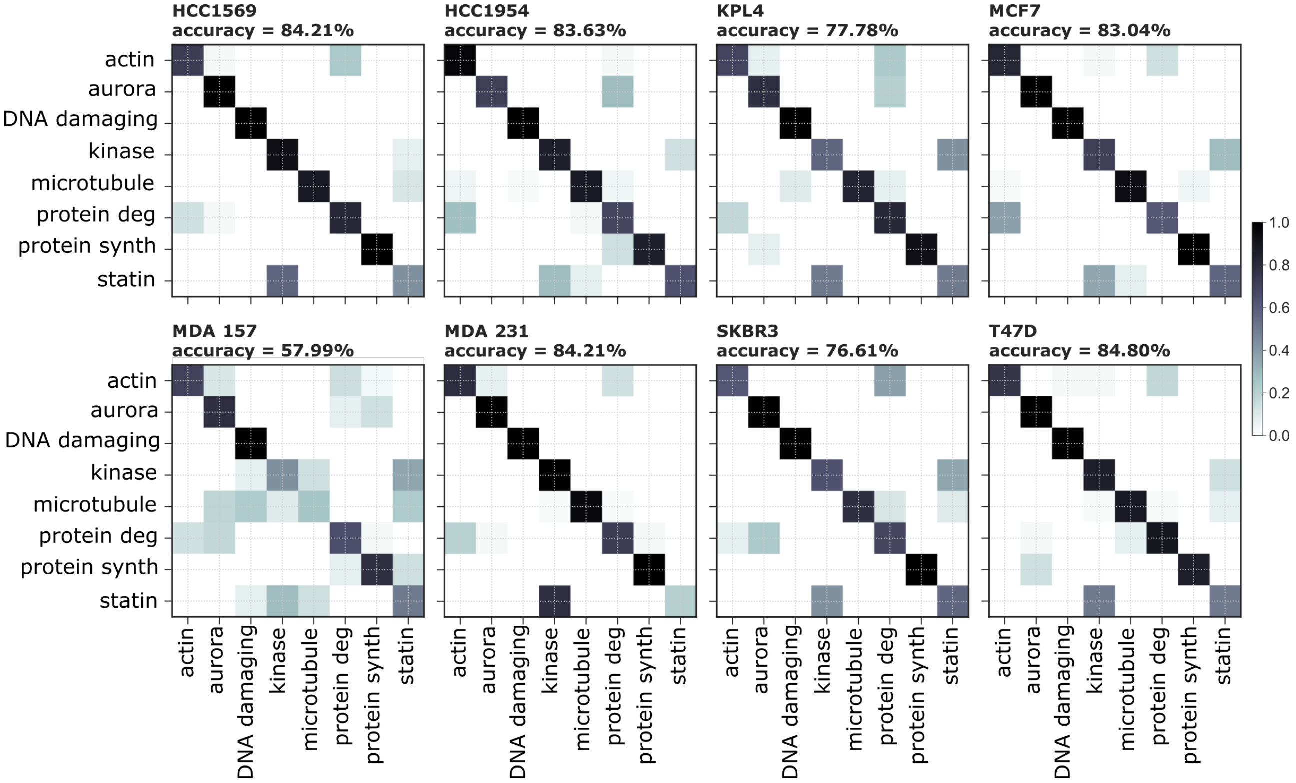

Figure S4: Confusion matrices for mechanism-of-action prediction using transfer learning of ResNet18 trained on seven cell-lines and trained on 10% of the unseen cell-line with six frozen convolutional layers and a reduced learning rate. Titles indicate the “unseen” cell-line.

Leave-One-Compound-Out Cross Validation

One of the issues with using a sampled training-test set is that a classifier can be trained and tested using examples of the same compound, such as replicates being split across training and test sets, or the trained classifier having been previously exposed to different concentrations of the same compound. This can lead to reported accuracies which are over-optimistic, and a classifier which might generalize more poorly than expected when used with a truly unseen compound.

Another evaluation strategy which has been used in the field is leave-one-compound-out (LOCO) cross validation, which creates a training set by excluding a compound from training (at all concentrations) which is then evaluated on the withheld compound. Here we have implemented a LOCO cross validation on a single cell-line, as well as a leave-one-compound-and-cell-line-out (LOCACLO) out cross validation, in which a model is trained in the absence of a specified compound and cell-line, and then evaluated on the unseen compound and cell-line. As this method generates many combinations of cell-line and compound pairs we only assessed LOCACLO using a single unseen cell-line (MDA-MB-231) for the computationally expensive CNN models.

Methods

LOCO and LOCACLO cross validation was implemented for both ResNet18 and gradient-boosted tree models by iterating through all 24 tested compounds, and for each compound excluding the data for that compound at all concentrations from the training set, and training the classifiers to minimize errors at predicting MoA classes as described in the main text methods section. For the LOCO models the classifier was then tasked with predicting the MoA class of the withheld compound in the same cell-line it was trained in. For the LOCACLO models, the classifier was trained on data excluding an entire cell-line as well as excluding data for the specified compound in the remaining seven cell-lines; then tasked with predicting the MoA class for the specified compound in the unseen cell-line.

Training data was balanced to account for different numbers of training samples in each group by under-sampling over-represented classes, and consensus classification and reported accuracies were calculated as per the main text methods.

Results

As would be expected, using the LOCO and LOCACLO cross-validation strategies decrease prediction accuracies when compared to training and testing on random partitions of image-data for both CNN and gradient boosted tree classifiers.

In figure S5 we see that these cross-validation strategies mirror the results in the main text in that there was a significant loss of prediction accuracy when the CNN model was transferred to an unseen cell-line.

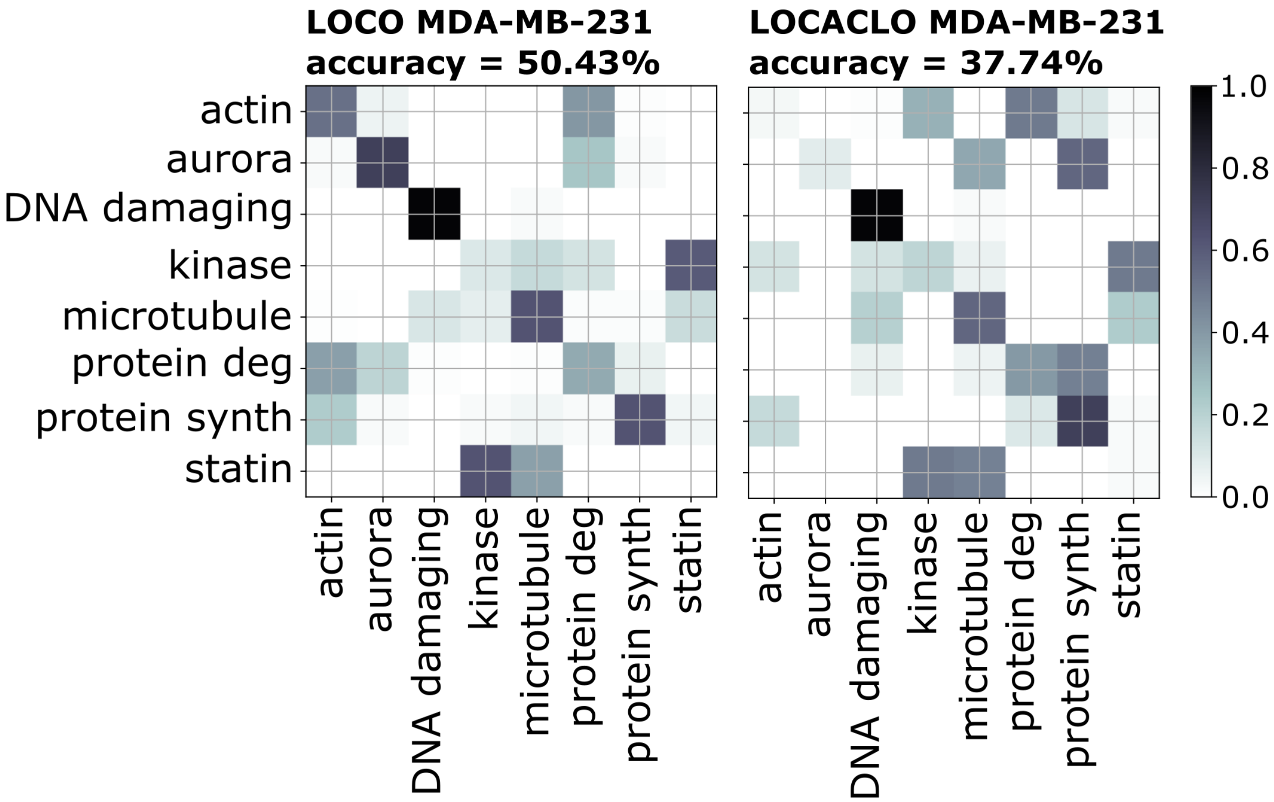

Figure S5: Confusion matrices for mechanism-of-action prediction using leave-one-compound-out (LOCO) and leave-one-compound-and-cell-line-out (LOCACLO) using CNN classifiers. LOCO (left) shows the confusion matrix when training and testing on the MDA-MB-231 cell-line. LOCACLO (right) shows the confusion matrix when training and on seven other cell-lines and testing on the unseen MDA-MB-231 cell-line.

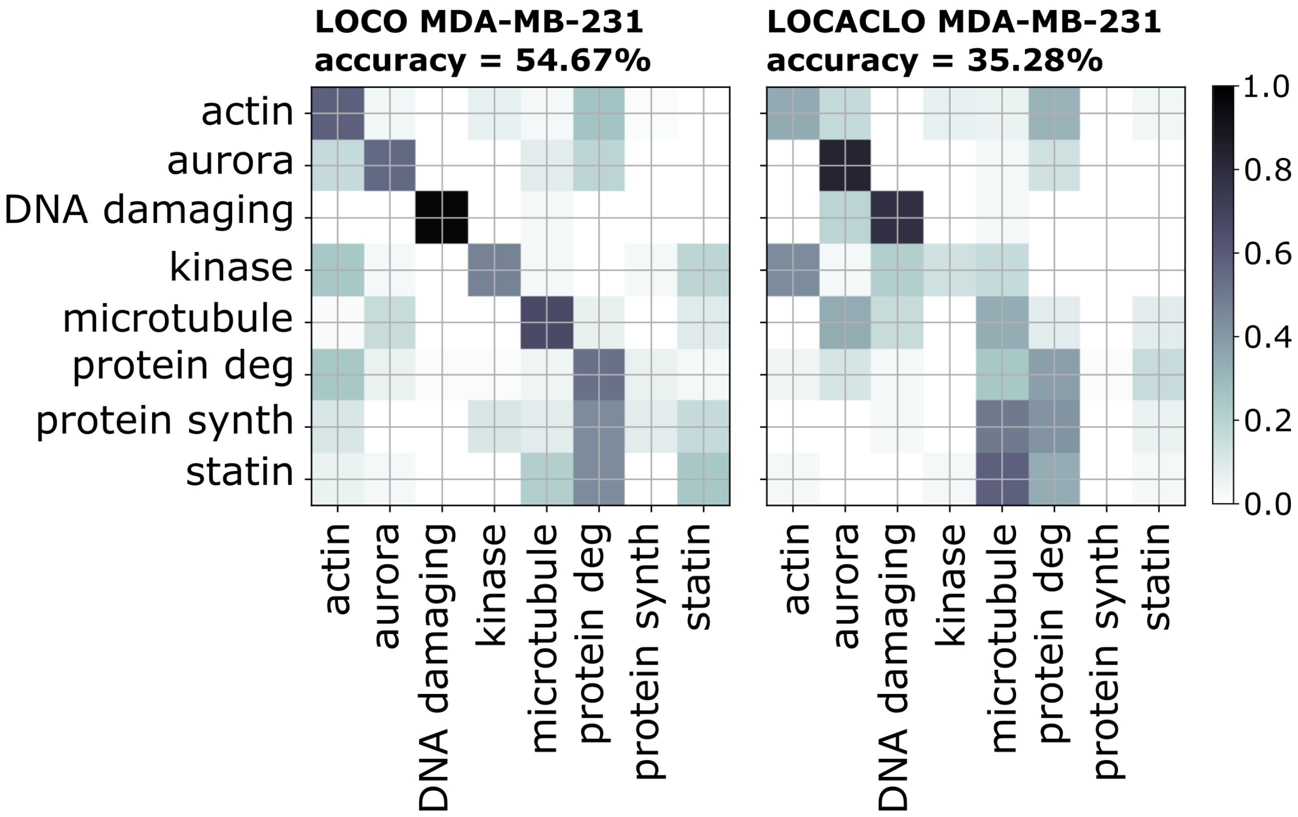

Figure S6: Confusion matrices for mechanism-of-action prediction using leave-one-compound-out (LOCO) and leave-one-compound-and-cell-line (LOCACLO) using gradient boosted tree classifiers. LOCO (left) shows the confusion matrix when training and testing on the MDA-MB-231 cell-line. LOCACLO (right) shows the confusion matrix when training on seven other cell-lines and testing on the unseen MDA-MB-231 cell-line.
